# Understanding catalysis in a multiphasic two-dimensional transition metal dichalcogenide

**DOI:** 10.1038/ncomms9311

**Published:** 2015-10-07

**Authors:** Stanley S. Chou, Na Sai, Ping Lu, Eric N. Coker, Sheng Liu, Kateryna Artyushkova, Ting S. Luk, Bryan Kaehr, C. Jeffrey Brinker

**Affiliations:** 1 Advanced Materials Laboratory, Sandia National Laboratories, Albuquerque, New Mexico 87106, USA; 2 Department of Physics, The University of Texas at Austin, Austin, Texas 78712, USA; 3 Department of Materials Characterization & Performance, Sandia National Laboratories, Albuquerque, New Mexico 87123, USA; 4 Center For Integrated Nanotechnologies (CINT), Sandia National Laboratories, Albuquerque, New Mexico 87123, USA; 5 Department of Chemical and Biological Engineering, The University of New Mexico, Albuquerque, New Mexico 87131, USA

## Abstract

Establishing processing–structure–property relationships for monolayer materials is crucial for a range of applications spanning optics, catalysis, electronics and energy. Presently, for molybdenum disulfide, a promising catalyst for artificial photosynthesis, considerable debate surrounds the structure/property relationships of its various allotropes. Here we unambiguously solve the structure of molybdenum disulfide monolayers using high-resolution transmission electron microscopy supported by density functional theory and show lithium intercalation to direct a preferential transformation of the basal plane from 2H (trigonal prismatic) to 1T′ (clustered Mo). These changes alter the energetics of molybdenum disulfide interactions with hydrogen (Δ*G*_H_), and, with respect to catalysis, the 1T′ transformation renders the normally inert basal plane amenable towards hydrogen adsorption and hydrogen evolution. Indeed, we show basal plane activation of 1T′ molybdenum disulfide and a lowering of Δ*G*_H_ from +1.6 eV for 2H to +0.18 eV for 1T′, comparable to 2H molybdenum disulfide edges on Au(111), one of the most active hydrogen evolution catalysts known.

## Abstract (web summary)

Establishing structure–property relationships for catalytic materials is essential for optimization of performance. Here, the authors solve the structure of molybdenum disulfide monolayers, and probe the role of lithium intercalation and the subsequent effects on catalytic hydrogen activation.

## Graphical abstract

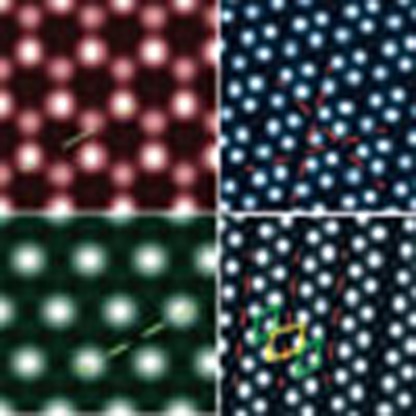

Improving the capacity and efficiency of the Hydrogen Evolution Reaction (HER) is an enduring challenge of green energy production and artificial photosynthesis^1^,^2^. Still, while HER in organisms evolved with time and increasing complexity, artificial HER aims to replicate the same with minimalism and simplicity. A cornerstone of the challenge is to mimic the function of natural hydrogenase enzymes, which catalyse HER in living systems. Indeed, it can be seen that without a catalyst like hydrogenase, HER does not proceed with the speed required for practical applications^3^,^4^. However, hydrogenases can be difficult to extract and purify, and can denature under non-natural operations^5^, consequently, inorganic alternatives are used for most applications. The most common example of this is platinum (Pt), which has served as the benchmark catalyst for HER due to its high catalytic efficiency^6^,^7^,^8^. Nevertheless, because of the scarcity and cost of Pt, a more abundant alternative is needed for cost-effective implementation.

For this, MoS_2_, an earth abundant lamellar solid, has shown prominent HER catalysis nearing the efficiency of platinum^9^,^10^. However, experiments using MoS_2_ grown on Au(111) indicated that this material is only catalytic on its edge sites^9^. Theoretical studies corroborated these results with the Gibbs free energy of hydrogen adsorption (Δ*G*_H_), a measure of HER efficiency, to be feasible for catalysis only at MoS_2_ edges^11^,^12^; the basal plane of MoS_2_ does not appear to participate in catalysis, meaning the bulk of material is catalytically inert. Consequently, the maximization of MoS_2_ edges and mimicry of the edge structure has become a significant topic^13^,^14^,^15^,^16^.

Interestingly, recent studies have begun to show enhancement of MoS_2_ catalytic efficiency following lithium intercalation and exfoliation^17^,^18^,^19^. The Tafel-slope of these sheets, a benchmark of electrochemical efficiency against applied overpotential, is nearly twice that of natural MoS_2_ after lithium treatment^17^,^18^,^19^. As the lithium-exfoliation reactions increase the availability of basal plane surfaces but not edges, the catalytic improvements are postulated to be basal plane related. Nonetheless, because the basal plane structures of lithium-exfoliated monolayers, and indeed, many two-dimensional sheets, are difficult to characterize^20^,^21^,^22^, the post lithiation and exfoliation structure of MoS_2_ remains nebulous and historically controversial, rendering the origin of this catalytic enhancement correlatively vague. To briefly recount a history of the structural understanding of MoS_2_ exfoliated with the assistance of lithium, it can be seen that a structural change in Li-intercalated MoS_2_ has been reported as far back as 1973 (ref. 23), nevertheless, interpretations of the final structure vary significantly in the literature. For example, in 1973, Somoano *et al*.^23^, observed extensive layer displacements after intercalation, suggesting the resulting product is a mixture of disordered compounds. However, in 1983, Py *et al*.^24^ reported the alkali metal exfoliated structure to be crystalline, with a first order phase transition from the natural trigonal prismatic (2H) to an octahedral (1T) phase. In 1989, a significant distortion to a 2a_o_ × 2a_o_ lattice was reported by Chrissafis *et al*.^25^ In 1991, an octahedral structure was observed by Jimenez *et al*. and Qin *et al*., but a small distortion was noted, making the final crystal a 2a_o_ × a_o_ super lattice^26^,^27^. Atomic force microscopy images obtained in 1993, by Schumacher *et al*., though, indicated no distortion or superlattice^28^. In 1998, Dungey *et al*. observed a 2a_o_ × 2a_o_ lattice with trigonal Mo clustering^29^, but in 1999, a 2a_o_ × a_o_ superlattice was seen by Heising *et al*., with severe distortion and formation of infinite Mo-zig-zag chains^30^. A chain-of-diamonds motif was suggested by Petkov *et al*. in 2002 (ref. 31), but most recently, in 2013, Maitra *et al*. reported a distortion-free octahedral phase^32^. Chhowalla and coworkers observed similar variations, from a perfect octahedral phase with natural 2H MoS_2_ domains^33^, distrted phases^19^, coexistence of various phases^34^, and also, a perfect octahedral phase^35^. Surprisingly, we note that the changed structures are almost always referred to as 1T, regardless of polymorphic structural differences. It is therefore not surprising to see recent catalytic improvement being attributed to varied structures, including octahedral^17^,^18^,^32^, and distorted Mo phases^36^,^37^. Part of the confusion is likely a simple consequence of incomplete transformation resulting in identification of partially transformed and transitional states due to variations between batches, however, there may be underlying issues of stability as well, making the resultant structure, and indeed, structural based predictions of its catalytic effectiveness difficult to handle^38^.

To address these long-standing issues and shed light on the catalytic origin of the transformed crystals, here we take a combined experimental/theoretical approach using controlled processing conditions to achieve stable phases and density functional theory calculations to investigate the stability of these polymorphs. With the solution processing advantages of these materials^39^,^40^,^41^, we report the catalytic efficiency of HER in homogenous reactions, with experimental H_2_ yield of these phases, and corroborate them with calculated Δ*G*_H_. The analysis was then extended from MoS_2_ to WS_2_ to show similarly distorted crystal phases and basal plane catalytic activation.

## Results

### Exfoliation and phase transformation of MoS_2_

A typical lithium-exfoliation reaction consisted of immersing MoS_2_ powder in *n*-butyl lithium (1 M) for 72 h at room temperature^31^,^33^,^42^. The intercalated compound was then transferred to water and sonicated to yield exfoliated monolayers. After purification using centrifugation and dialysis, concentrations and purity of each batch were measured using Flame Atomic Absorption Spectrophotometry. Samples were then visualized using abberation-corrected scanning transmission electron microscopy.

**Figure 1:**
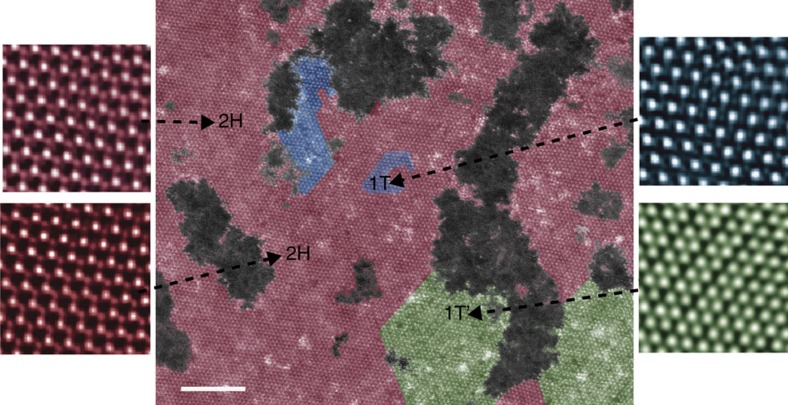
Polymorphism and incomplete transformations. Polymorphisms within the basal plane exhibit coexistence of three different phases, 2H (trigonal prismatic, false coloured as red), 1T (octahedral, false coloured as blue) and 1T′ (clustered Mo, false coloured as green). Scale bar, 5 nm.

As seen in Fig. 1, a typical sheet exfoliated using this method indeed yields a mixed phase (shown without false colouring in Supplementary Fig. 1). First, a trigonal prismatic (2H) phase that corresponds with an untransformed basal plane can be seen with symmetrical Mo–Mo spacing of 2.98±0.05 Å. Second, an octahedral phase with displaced sulfur atoms and symmetrical Mo arrangements (2.95±0.06 Å) is seen, albeit in small quantities. Last, large swatches of a visually distinct, tertiary phase is also present, with Mo atoms asymmetrically clustered to form one-dimensional lines of alternating light-and dark stripes. In this phase, Mo–Mo distances of 3.55±0.16 Å and 2.92±0.16 Å were measured. On the basis of the above, we thus conclude that within one individual sheet, a microcosm of various phases are present, with each representing differing interpretations in previous work. Nevertheless, as the stability of each phase should be an intrinsic consequence of the energetics of distinct atomic arrangements, we sought to understand the relative stabilities of each phase. Because it was previously reported that materials revert back to the natural 2H phase^42^, we reasoned that the 2H is the overall energetic minimum, with the other polymorphs occupying differing metastable points in relation to the 2H.

To better define this, we employed Density Functional Theory (DFT) to independently predict the structures of possible polymorphs (see Supplementary Information for computational details). As can be seen in Table 1, a total of four polymorphs were predicted based on structural optimization of unit cells. The first two, consisting of 1 × 1 unit cells, were the 2H trigonal prismatic phase and the 1T octahedral phase. In addition, a distorted phase with zig-zag chains consisting of a 2 × 1 supercell and a phase with Mo–Mo atoms clustered into trimerized pockets in a 2 × 2 supercell were predicted. We dub the two later phases, 1T′ and 1T′′, respectively.

Next, the stabilities of each DFT polymorph were calculated. It can be seen that the symmetrical 1T representation has the least stable ground-state (+0.82 eV versus 2H). Indeed, in a 2 × 2 or 4 × 4 supercell, we find the 1T phase eventually relaxes into 1T′′, which is second in stability, at +0.63 eV, conforming with the dynamical phonon instability previously predicted in the 1T phase^38^. Nevertheless, the 1T′ phase was significantly more stable than 1T and 1T′′ (+0.55 eV versus 2H), with a barrier against 2H reversion of 0.73 eV f.u.^−1^ (formula unit, Supplementary Fig. 2, Supplementary Table 1). By the DFT calculations, the transformed portions within a sample, if allowed to reach metaequilibria, should therefore preferentially form 1T′ instead of the other polymorphs.

**Figure 2:**
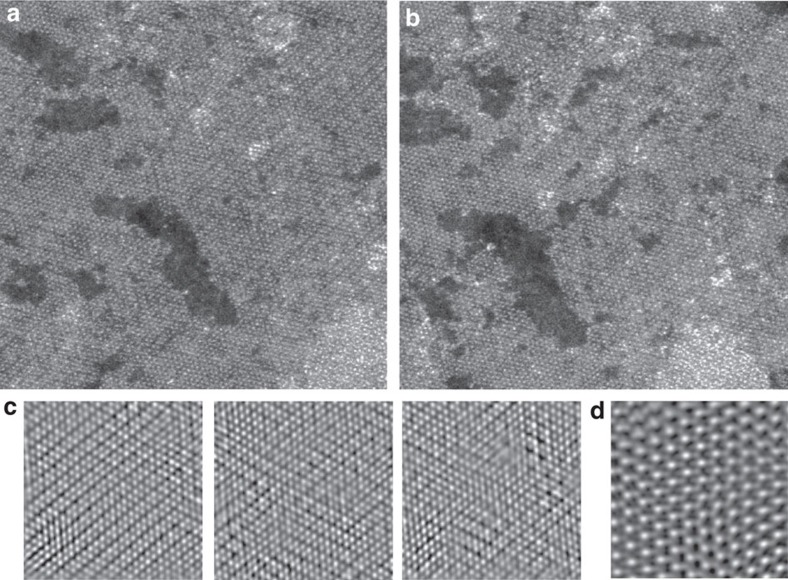
Basal plane transformations. (**a**) MoS_2_ basal plane showing preferential transformation to 1T′ with increased lithiation, in accordance with the calculated relative stability of the metastable phases. (**b**) On electron beam illumination, the basal plane exhibits a restoration to a symmetrical Mo arrangement indicative of 2H reversion. (**c**) Sequential frames taken 60 s apart, showing phase evolution under the electron beam. Electron dose rate was 2,800 e Å^−2^ s^−1^, with each frame requiring 40 s to capture. (**d**) An electron dosed region with sulfur atoms resolved, demonstrating 1T′ to 2H phase reversion after illumination.

To investigate these results experimentally, we prepared samples to induce equilibria by extending the lithium intercalation period. As seen in Fig. 2a, a preferential transformation to 1T′ is possible on the entirety of the basal plane. This therefore suggests that the various polymorphs observed previously are likely consequences of incomplete reactions, such as partial intercalation, with parts of the basal plane yet to undergo sufficient conversion to the 1T′. Indeed, we have observed that when intercalation times are extended from 72 to 240 h, a complete transformation to the 1T′ can be achieved (Supplementary Fig. 3).

Given the DFT prediction of 1T′ as a metastable phase, we examined the ability to covert 1T′ back to 2H under electron beam-illumination. As shown in Fig. 2c, sequential frames taken at 60 s intervals (acquired at 40 s/frame at an electron dose rate of 2,800 electron Å^−2^ s^−1^) show gradual relaxation of Mo atoms from 1T′ lines to symmetrical 2H spacing. A detailed investigation of the structures again showed the 1T′ phase with Mo spacings of 3.55 Å and 2.92 Å, and the relaxed structure displayed trigonal prismatically coordinated sulfur atoms with Mo–Mo spacing of 2.95 Å, in agreement with the 2H structure (Fig. 2d). These results corroborate the 1T′ as the energetically preferred metastable phase that will relax back to 2H when energetic input, for example, heat, exceeds that of the metastable barrier energy. It further suggests that the partial or incomplete phase transformations observed previously may be due to energetic conditions reverting the material to 2H during the characterization process. Supplementary Fig. 4 shows the final product of 1T′ heated under inert atmosphere is a 2H lattice. These observations support previous electrical characterizations of the material^33^,^42^ with 1T′ having greatly reduced resistivity compared to 2H (calculated bandgap of 0.01 and 1.7 eV, respectively, shown in Table 1).

**Figure 3:**
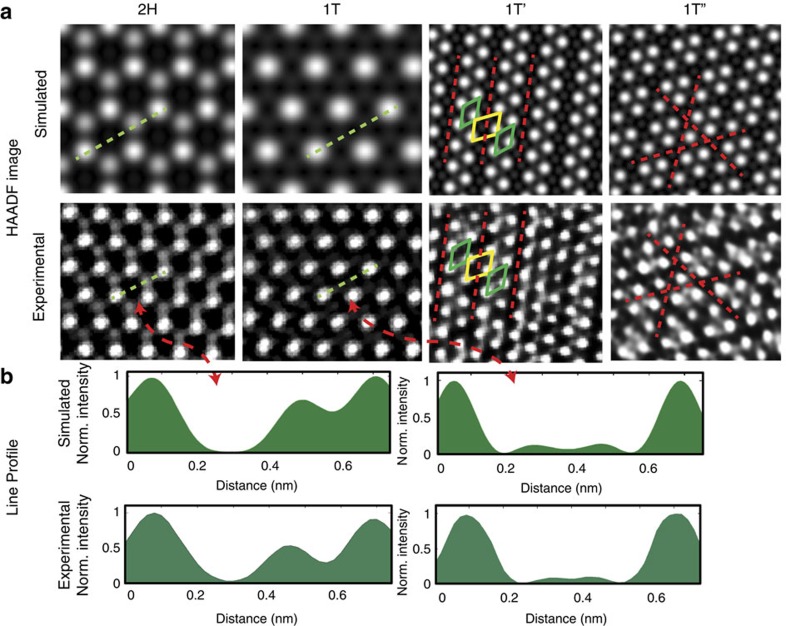
Different possible phases for MoS_2_. (**a**) Simulated and experimental images of different possible phases for MoS_2_. FFT mask filters have been employed for clarity. (**b**) line scan comparisons between 2H and 1T using the simulated and experimental images, showing modulation of sulfur contrast.

To corroborate the above, High-angle annular Dark-field (HAADF) images were simulated using coordinates from DFT. As seen in Fig. 3, good agreement was seen between experiment and simulations. We note in samples that are predominantly 1T′ (Fig. 2a), lines modulated in two-dimensions can be seen at intersections of 1T′. This may be interpreted as the 1T′′, however, its occurrence is very localized and defective (<5%), and consequently, not of practical significance.

### Dye-sensitized HER

Following these structural insights, HER catalysis of these MoS_2_ polymorphs was examined. For this, we measured H_2_ formation using MoS_2_ in a homogenous photocatalysis reaction. In a typical reaction, a solution of MoS_2_ was sensitized with Eosin Y (EY), and Triethanolamine (TEOA) was added as a sacrificial electron donor^43^,^44^. The reaction mixture was then purged of atmospheric gasses, and irradiated with a xenon lamp tuned to 1 sun. The flow entering and exiting the reaction flask was then monitored on a gas chromatograph to quantify H_2_ formation inside the flask.

**Figure 4:**
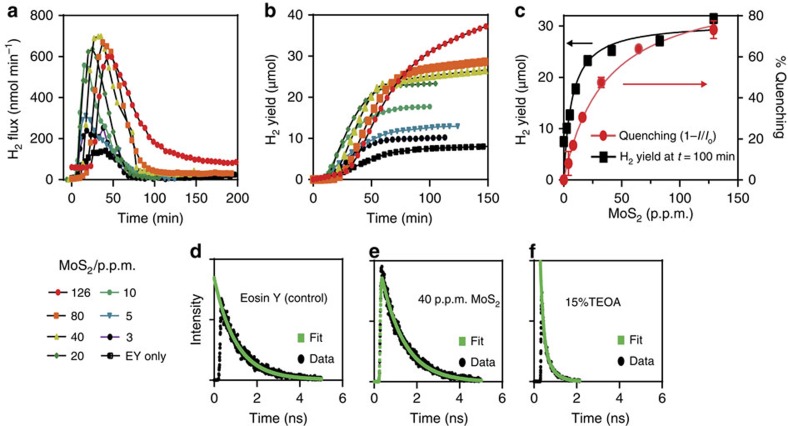
Hydrogen evolution. With dye sensitization (eosin Y, EY), MoS_2_ can be used to conduct homogenous hydrogen evolution reactions in solution. (**a**) Real time H_2_ flux at incremental MoS_2_ concentrations. Diminished reactions are indicative of proton exhaustion, and can be restarted with acid addition. (**b**) Corresponding cumulative H_2_ yield (For **a** and **b** note key bottom left). (**c**) Incremental concentration of MoS_2_ and correlative overlay of cumulative H_2_ yield (black) with fluorescence quenching of dye (red) in solution. (**d**) fluorescence decay of eosin Y, (**e**) reaction saturated with eosin Y and MoS_2_ showing similar decay, indicative of static-quenching and ground-state complex, and (**f**) collisional quenching between TEOA and eosin Y resulting in second order decay and attenuated lifetime. Error bars denote s.d.

The reaction proceeded via photoexcitation of EY and subsequent intersystem crossing to yield a triplet excited state (EY^3^*). Acceptance of an electron via reductive quenching from the sacrificial electron donation (TEOA) then yields a radical EY^−^32,43,45,46,47. The highly reductive EY^−^ can then reduce a proton to form H_0_, return the electron to an oxidized TEOA, or transfer the electron to another catalyst such as the MoS_2_. As seen in Fig. 4a, in the absence of MoS_2_, direct reduction of protons by radicalized EY occurs by collisional reductive quenching with TEOA (Supplementary Fig. 5), and generates a peak H_2_ flux of 150 nmol min^−1^. Over the course of 100 min, a yield of 7.5 μmols was observed, giving a typical activity of 4.5 μmol h^−1^ (Fig. 4b). Sequential addition of 1T′ (for example, ‘fully transformed MoS_2_') gradually raised the peak flux, until saturation was reached with 40 p.p.m. of MoS_2_, yielding a peak flux of 620 nmol min^−1^. With the latter reaction, 40 p.p.m. of MoS_2_ raised overall yield to 30 μmol in the first 100 min, giving a typical activity of 18 μmol h^−1^, which is four times higher than the autocatalytic EY dye under the same conditions^43^. In both cases, addition of small amounts of HCl regenerates a stopped reaction, suggesting reaction termination is due to proton depletion (Supplementary Fig. 6).

Given the boosted HER performance, it reasons that in the presence of fully transformed MoS_2_ a preferential charge transfer occurred between the radical EY^−^ and the MoS_2_. Indeed, with the reductive potential of EY at −0.8 V versus normal hydrogen electrode (NHE), and MoS_2_'s conduction band situated at 0.2 V versus NHE, such a transfer is feasible^48^,^49^. To support this, the charge transfer was examined using fluorescence quenching correlations between EY and the Li-exfoliated MoS_2_ (Fig. 4c). It can be seen that the fluorescence quenching interactions between EY and MoS_2_ are predictive of the eventual H_2_ yield, with both curves rising to saturation in the presence of 40 p.p.m. of MoS_2_. The fluorescence quenching following a saturation reaction fitting, vis-à-vis a continual diminishing of fluorescence with increasing quencher concentration (for example, collisional quenching) indicates the formation of a ground-state complex between EY and lithium-exfoliated MoS_2_, facilitating effectual charge transfer. To further elucidate these interactions, we monitored the excited state interactions via fluorescence decay. As seen in Fig. 4d, the fluorescence lifetime of a singlet excited EY, in the absence of secondary interactions, is ∼1 ns. However, interactions with reductive quenchers, such as TEOA, via collisional quenching results in fluorescence lifetime shortening that fits a two exponential decay (Fig. 4e)^50^. Interactions between EY and MoS_2_ did not follow this behavior. Instead, the lifetime of EY was maintained at ∼ 1 ns (Fig. 4f), while the fluorescence intensity was attenuated by 75% at saturation. This indicates adsorption of EY onto MoS_2_ to form a non-fluorescent ground-state complex that facilitates charge transfer within the complex with an excited state lifetime following the behavior of the 25% minority free dyes^50^.

**Figure 5:**
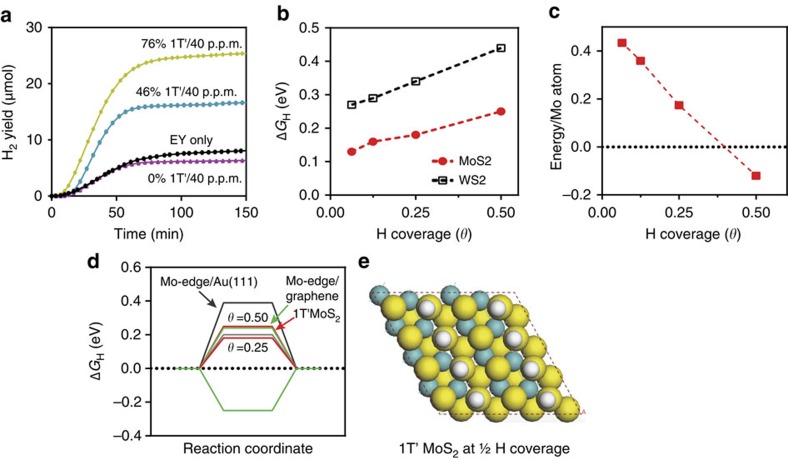
Energetics of Hydrogen Evolution. (**a**) Cumulative H_2_ yield with MoS_2_ undergoing differing degrees of 1T′ transformation. (**b**) DFT calculated Gibbs free energy of hydrogen adsorption (Δ*G*_H_) as a function of H coverage. (**c**) DFT calculated stability of the MoS_2_ structures per Mo atoms as a function of H coverage. As the H coverage exceeds 0.4, the stability of the 1T′ phase (red dashes) surpasses that of the 2H (black dots). (**d**) Free-energy diagram of hydrogen evolution reaction at 1/2 and 1/4 H coverage, with 1T′ MoS_2_ basal plane, 2H MoS_2_ edge on graphene and 2H MoS_2_ on Au(111) as reference. (**e**) Structural representation of 1T′ at 1/2 H coverage.

As the above were performed with the fully transformed 1T′, effect of partial 1T′ transformation was then investigated. Here, two additional MoS_2_ batches with diminishing 1T′ transformation were used in identical reactions. As shown in Fig. 5a, the MoS_2_ batches with diminished 1T′ transformation yielded significantly less H_2_. Indeed, it can be seen that H_2_ yield scaled proportionately with 1T′ signatures, which dominates the basal plane of the exfoliated sheets (Supplementary Fig. 7, Supplementary Table 2). Given that 1T′ has shown empirical evidence of improved electrical conductivity, it is therefore possible that the improved catalysis seen with the ‘fully transformed MoS_2_' is a consequence of better electron conduction from the EY^−^ charge transfer site to the catalytic sites of MoS_2_. However, it is also possible that the 1T′ transformation fundamentally alters the catalytic mechanism of the MoS_2_ sheets, possibly giving additional active sites for catalysis.

To investigate, we calculated, via DFT, the free energy of hydrogen adsorption (Δ*G*_H_), for the basal plane of each polymorph. From previous reports, it is known that |Δ*G*_H_|≈0 describes an optimal adsorption condition for catalysis^10^. Significant deviation in the exothermic direction (Δ*G*_H_<0) can indicate irreversible adsorption, consequently, an efficient catalyst such as the 2H MoS_2_ edge site, tends towards being slightly endothermic (Δ*G*_H_>0). From our calculations, Δ*G*_H_ for the 2H basal plane is >+1.6 eV regardless of the H coverage and the absorption site. As a result, the adsorption of H on the 2H basal plane is strongly unfavorable. This is consistent with the 2H surface being catalytically inert^9^. However, when the basal plane is converted to 1T′, the hydrogen binding energy becomes negative (adsorption occurs). As seen in Fig. 5b, the free energy of adsorption here for 1T′ MoS_2_ is reasonably close to the optimum value of ≳0 eV (ref. 12), emerging at ΔG_H_=0.13 eV at 1/16 coverage and growing to 0.25 eV as H coverage increased to 1/2. This indicates that the 1T′ transformed surface is amenable for catalysis, and indeed, the HER improvements with 1T′ fraction is due to catalytic activation of the MoS_2_ basal plane. Most interestingly, we also observe adsorption of hydrogen to stabilize the 1T′. Indeed, Fig. 5c shows that when H coverage exceeds 0.4, 1T′ becomes more stable than 2H. As significant benchmark work with 2H MoS_2_ was performed with Au (111) supports^9^, we compare 1T′ with 2H Mo-edge sites, when supported on gold. It can be seen here that |Δ*G*_H_| on the 1T′ basal plane is comparable to the 2H Mo-edge supported on Au (111) and graphene at 0.25 coverage and slightly outperforms Mo-edges on Au (111) at 0.5 coverage (Fig. 5d,e)^51^. Above all, the shear increase in surface area availability after exfoliation to monolayers (100–1,000-fold greater than bulk solids)^52^ renders the basal plane improvements even more significant.

As WS_2_ can undergo analogous phase transformations, its catalytic efficiency was similarly analyzed. We show in Supplementary Fig. 8, the H_2_ flux with WS_2_ was a third lower than MoS_2_. For peak H_2_ flux, WS_2_'s 400 nmol min^−1^ showed approximately threefold improvement over controls, but weaker than the 750 nmol min^−1^ observed for MoS_2_. Similarly, H_2_ yield with WS_2_ was 18 μmol at *t*=100 min, which is 33% lower than MoS_2_. As fluorescence lifetime measurements revealed an analogous molecular charge transfer pathway, the reduced HER efficiency was thus indicative of WS_2_ being a less efficient catalyst. From Fig. 5b, it can be seen that the Δ*G*_H_ of 1T′ transformed WS_2_ was 0.15 to 0.2 eV higher than MoS_2_. Nevertheless, this value represents a 10-fold reduction over Δ*G*_H_ of 2H WS_2_, indicating catalytic activation of the basal plane comparable to MoS_2_.

## Discussion

We investigated the structure of MoS_2_ exfoliated with lithiation intercalation and directly correlated the varying physical structures to the catalytic origin for HER. Particularly, we demonstrated via abberation-corrected scanning transmission electron microscopy that modulated lithiation can lead to complete 1T′ transformation in the basal plane, and corroborated the experimental findings with DFT calculated phase stabilities. It was shown that the Mo atoms of MoS_2_ became asymmetrically spaced with increased lithiation, thus forming the 1T′ phase. In DFT, the 1T′ was shown to be energetically favorable compared to other possible polymorphs. Nevertheless, external perturbations within the environment, including electron imaging parameters, can induce a reversion to 2H.

With regard to catalysis, we have shown that the 1T′ transformation rendered the normally inert basal plane amenable towards hydrogen adsorption and H_2_ evolution. Indeed, ΔG_H_ on the basal plane of 1T′ MoS_2_ showed catalytic activation and was lowered from +1.6 eV in the 2H to +0.18 eV. Moreover, when H coverage became >0.4, DFT showed 1T′ phase stability surpasses that of 2H. To put this in perspective, the ΔG_H_ of 0.18 eV is comparable to 2H MoS_2_ edges on Au(111), one of the most active HER catalysts yet characterized. This makes the 1T′ MoS_2_ a state-of-the-art catalyst. In addition, as exfoliation to monolayers increases the surface area by as much as 1,000-fold^52^,^53^, the basal plane activation provides non-trivial increases in catalytic efficiency compared with the edge only catalysis of the 2H (refs 9, 15). We demonstrate this by H_2_ evolution studies in basic solution (pH 11), via self-assembled photocatalytic charge transfer complexes for HER. Here it can be seen that MoS_2_ catalysts can boost H_2_ yield of the HER catalytic dye, EY, by fourfold. Moreover, MoS_2_ itself does not fatigue over the course of the reaction. A clearer understanding of these materials, and the underlying relationship between structure, properties and performance, provides a pathway towards quantitative engineering of MX_2_ to enable the emerging ‘green energy' economy.

## Methods

### Lithium intercalation and exfoliation

For MoS_2_, lithium intercalation was accomplished by immersing 1 g of MoS_2_ powder in 10 ml of 1 M *n*-butyl lithium. The mixture was stirred vigorously in an inert atmosphere glovebox for 3 to 10 days. After, the compound was washed over three layers of Whatman filter paper (#51, ashless) with 200 ml of hexane and then collected in a bottle. Three hundred millilitres of H_2_O was then added, and the mixture was sonicated for 3 m to extricate the intercalated MoS_2_ powder from the filter paper. The filter paper was then removed from the bottle, and the solution was sonicated for an additional 1.5 h. Unexfoliated portions were removed with centrifugation at 100 *g* for 3 min. The supernatant was then collected, and purified by centrifugation and washing with H_2_O (3 × 10,000 g for 1.5 h, followed by resuspension in water after each centrifugation cycle). The solution was then transferred in to a dialysis bag (Fisher Scientific, MW 5,000), and dialysed against running water for 3 days. The solution was then again centrifuged at 500 *g* for 15 m to remove aggregates, and the resultant samples were used as is. For WS_2_, Li intercalation was performed at 100 °C in a Parr bomb. Exfoliation and purification of WS_2_ was the same as MoS_2_. Atomic force microscopy images of exfoliates sheets are shown in Supplementary Fig. 9.

### STEM microscopy and analysis

A FEI Titan G2 80–200 S/TEM with a Cs probe corrector operated at 200 kV was used in this study. High-angle annular dark-field (HAADF) S/TEM images were acquired with an electron probe size of 0.8 Å, convergence angle of 18.1 mrad, and current of ∼100 pA with an annular detector with a collection range of 60–160 mrad. The high resolution HAADF images were typically taken at 1,800 k magnification, yielding a pixel size of about 0.23 Å or a frame size of 48 × 48 nm for 2,048 × 2,048 pixels per frame. Such conditions gave rise to an equivalent electron dose rate of ∼2,800 electrons Å^−2^ s^−1^. Acquisition of the HAADF image (frame of 2,048 × 2,048 pixels) took ∼40 s at a dwell time of ∼10 μs/pixel. In sub-figures of Figs 1, 2 and 3, the filtered images were obtained by Fast Fourier transformation (FFT) of the HAADF images into reciprocal space, forming FFT patterns. Reciprocal spots from the patterns (typically {100} type reflections) were selected, masked with a 60 pixels filter, and then transformed into real space with inverse FFT. Gatan Digital Micrograph was used for the image processing. Additional details on TEM imaging and simulation are shown in Supplementary Methods

### Computational details

All density functional calculations were carried out using the Vienna *ab initio* simulation package^54^ with plane wave basis set and projector augmented-wave pseudopotentials^55^. All energies were calculated with Perdew–Burke–Ernzerhof exchange-correlation potentials^56^. For selected structures, we applied the hybrid HSE06 functional^57^ to calculate the electronic band gap. The MoX_2_ (X=Mo,W) monolayers were modeled using surface supercells separated in the periodic direction by a 20 Å-thick vacuum slab. We applied a plane wave energy cutoff of 600 eV and Γ-centered 24 × 24 × 1, 12 × 24 × 1, 12 × 12 × 1, and 6 × 6 × 1 k-points grids for Brillouin zone sampling of the 1 × 1 unit cell (2H and 1T), and the 2 × 1 (1T′), 2 × 2 (1T′'), and 4 × 4 (H/XS_2_) supercells, respectively. The criteria of convergence for energy and force were set as 10^−4^  eV and 3 × 10^−3^ eV/Å. The hydrogen adsorption energies were calculated using the 4 × 4 surface supercell containing 16 Mo atoms and 32 S atoms. A dipole correction was applied to cancel the electrostatic interaction between the periodic slabs. The DFT binding energies were calculated as 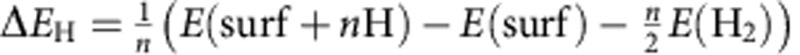, where E(surf+nH), E(surf), and E(H_2_) are the total energies for the MoS_2_ surface with *n* hydrogen atoms adsorbed, the clean MoS_2_ surface, and the molecular hydrogen in the gas phase, respectively. The most stable H binding site in the basal plane of MoS_2_ is on the top of the S atoms. We define H coverage as the ratio of the number of H and Mo atoms in the basal plane. For 1T′ MoS_2_, we obtained binding energies of −0.16 eV, −0.13 eV, and −0.11 eV, and −0.035 eV at the 1/16H, 1/8H, 1/4H, and 1/2H coverage. For the 4 × 4 supercell, this corresponds to *n*=1, 2, 4, and 8, respectively. The adsorption free energy was calculated by adding a thermal correction to the binding energy Δ*G*_H_=Δ*E*_H_+Δ*E*_ZPE_−*T*Δ*S*_H_, where Δ*E*_ZPE_ and *T*Δ*S*_H_ are, respectively, the differences in the zero point energy and entropy contribution between the H adsorbed state and H_2_ in the gas phase. We took ΔS_H_=−½ S(H_2_), where ½ S(H_2_) is the entropy of ½ H_2_ in the gas phase at standard conditions (*T*=298.15 K, Pressure=1 atm). We used the assumption that the vibrational entropy in the adsorbed state is small^58^. For H/1T′ MoS_2_, *E*_ZPE_=0.228 eV (vibrational frequencies 2,530.1 cm^−1^, 637.6 cm^−1^, 519.8 cm^−1^) and *E*_ZPE_=0.271 eV for H_2_. With these values, the Gibbs free energy was calculated as Δ*G*_H_=Δ*E*_H_+0.29 eV. Additional details are available in Supplementary Methods.

### Photocatalysis

Stock mixtures of 30% v/v TEOA and 10 mg ml^−1^ Eosin Y were freshly prepared for each reaction. MoS_2_ (and WS_2_) were prepared at twice the desired final concentration (for example, 80 p.p.m.). To prepare the reaction mixture, 35 ml of the 30% v/v TEOA was mixed with 35 ml of the MoS_2_ or WS_2_, reaching the final concentration of 15% v/v TEOA and the predetermined MoS_2_/WS_2_ concentration (for example, 40 p.p.m.). The mixture was then transferred to a 250 ml two-neck flask, to which 1 ml of the 10 mg ml^−1^ Eosin Y was added. The flask was then covered in aluminum foil, stirred and purged with continuous Ar flow (20 c.c.m.) for 20 min. After Ar purge, the aluminum foil covering was removed, and the mixture was illuminated at 1 sun. With the Ar flow kept at 20 c.c.m., gas samples were continuously monitored at 90 s intervals using an Inficon 3,000 micro GC gas analyzer until reaction termination.

## Additional information

**How to cite this article:** Chou, S. S. *et al*. Understanding catalysis in a multiphasic two-dimensional transition metal dichalcogenide. *Nat. Commun.* 6:8311 doi: 10.1038/ncomms9311 (2015).

## Supplementary Material

Supplementary Information Supplementary Figures 1-9, Supplementary Tables 1-2, Supplementary Methods and Supplementary References

## Figures and Tables

**Table 1:**
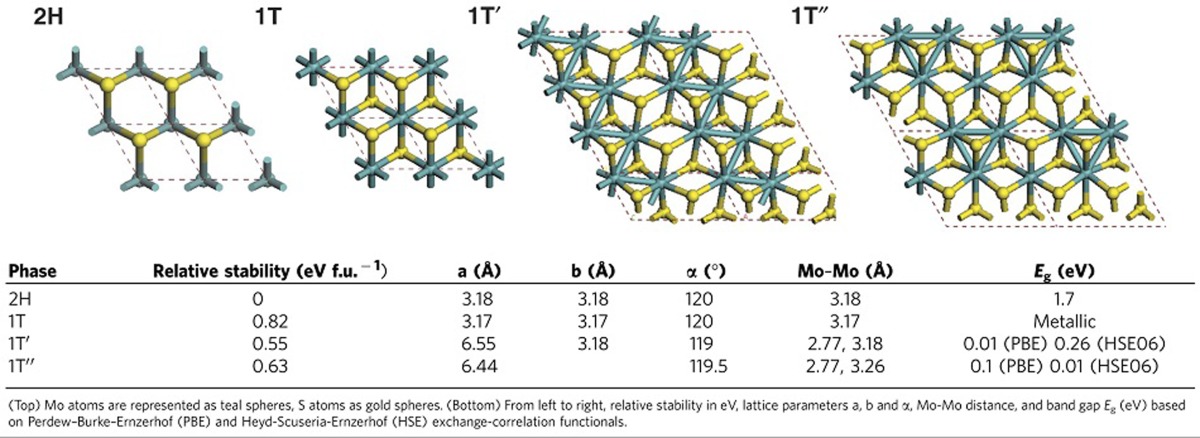
Structural polymorphs of MoS_2_ and summary of parameters as predicted by Density Functional Theory.
