# Supplementary material for: Understanding catalysis in a multiphasic two-dimensional transition metal dichalcogenide
Source: Nat Commun. 2015 Oct 7;6:8311. doi: 10.1038/ncomms9311 (PMC4633626; doi:10.1038/ncomms9311)
Supplement: Supplementary Information — Supplementary Figures 1-9, Supplementary Tables 1-2, Supplementary Methods and Supplementary References [file ncomms9311-s1.pdf]

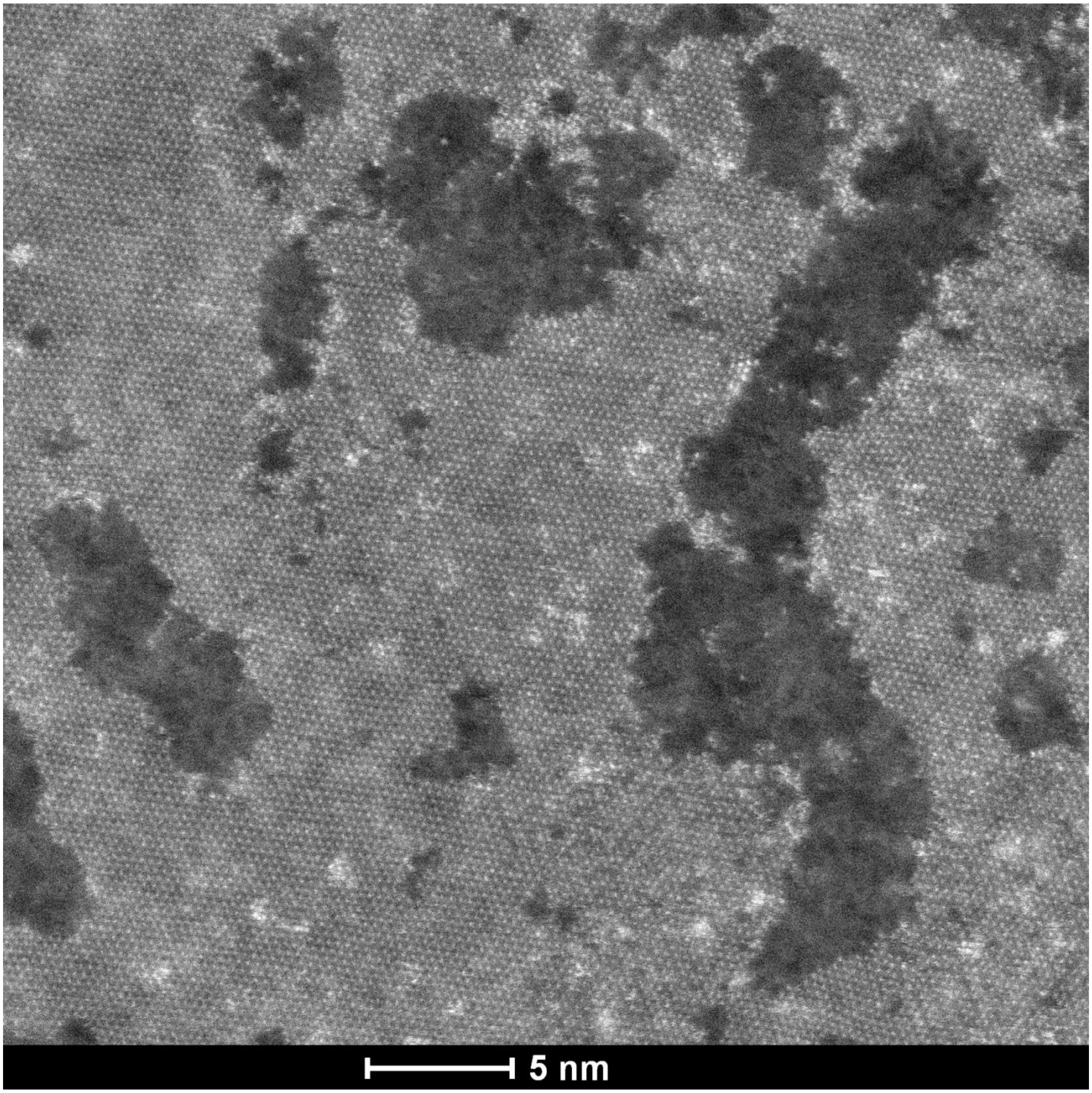

Supplementary Fig. 1. Exfoliated MoS_2 sheet shown in Figure 1, without false coloring

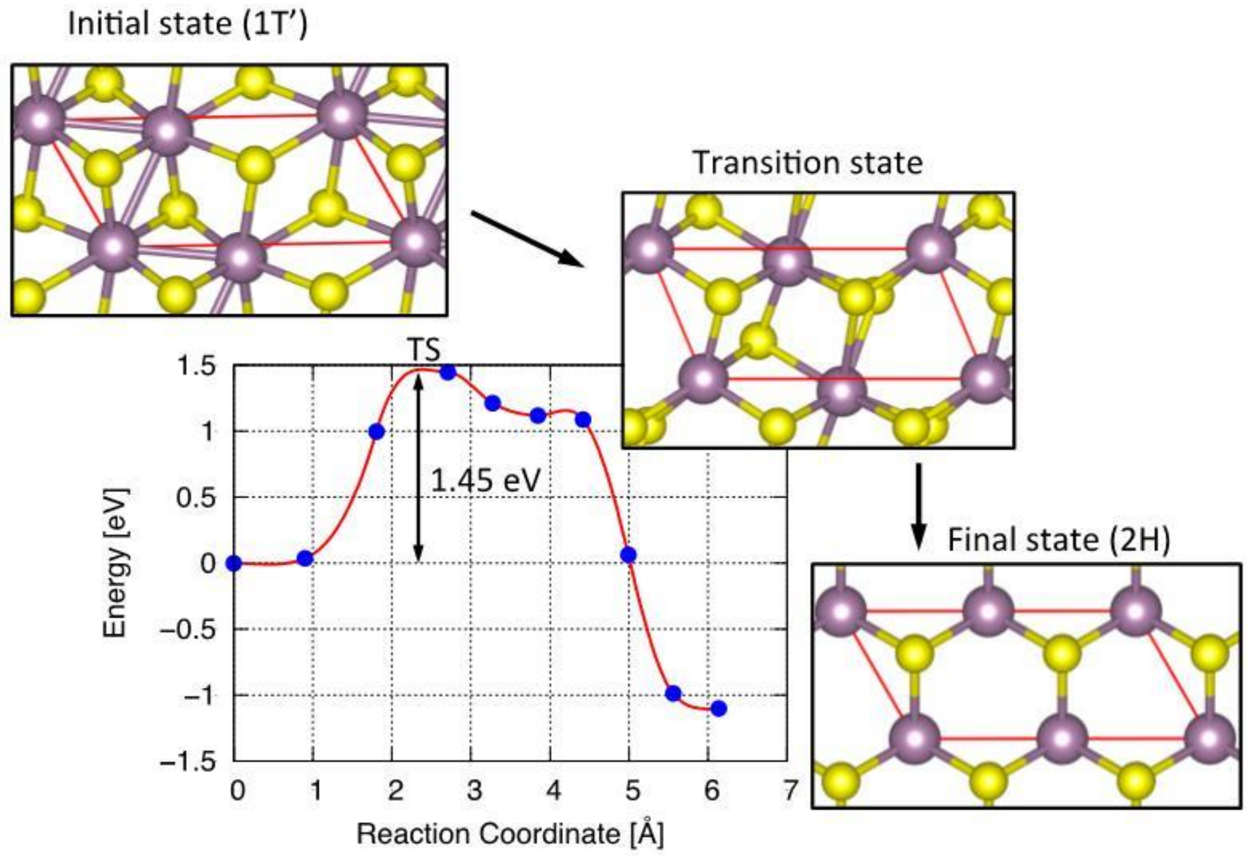

Supplementary Fig. 2. The minimum energy path along the transformation from the 1T' phase to 2H phase of MoS_2. The saddle point (transition state) has an activation energy barrier ΔE of 1.45 eV for a 2x1 cell, significantly higher than the stabilization energy of 1.10 eV of 2H (relative to 1T' in a 2x1 super cell). For one formula unit (f.u.), energy barrier ΔE is 0.73 eV/f.u. and stabilization energy is 0.55 eV/f.u.

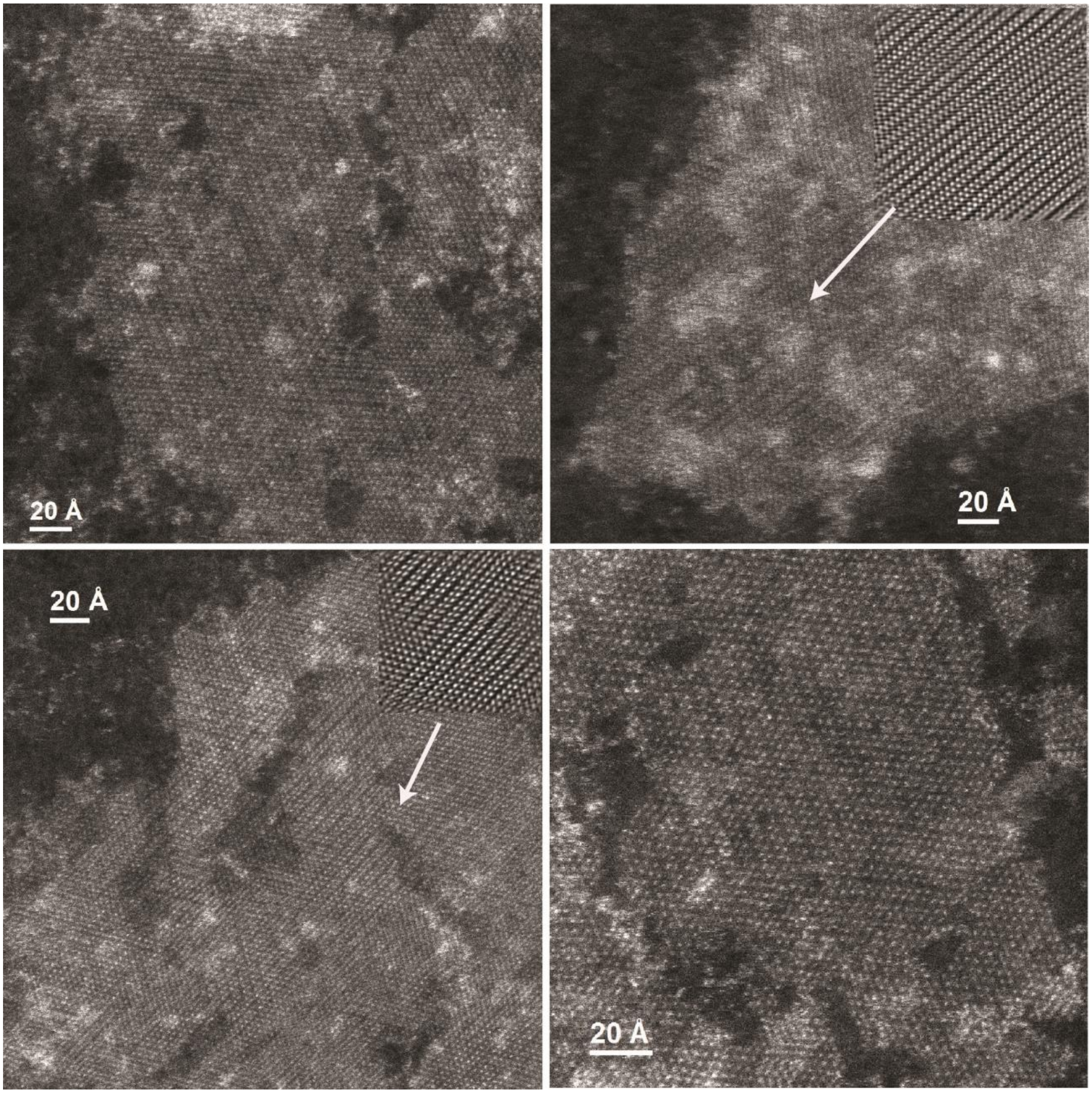

Supplementary Fig. 3. Additional TEM micrograph of MoS_2 sheets demonstrating complete conversion to 1T'. Specifically, striations characteristic of the 1T' can be seen covering the entirety of the basal plane.

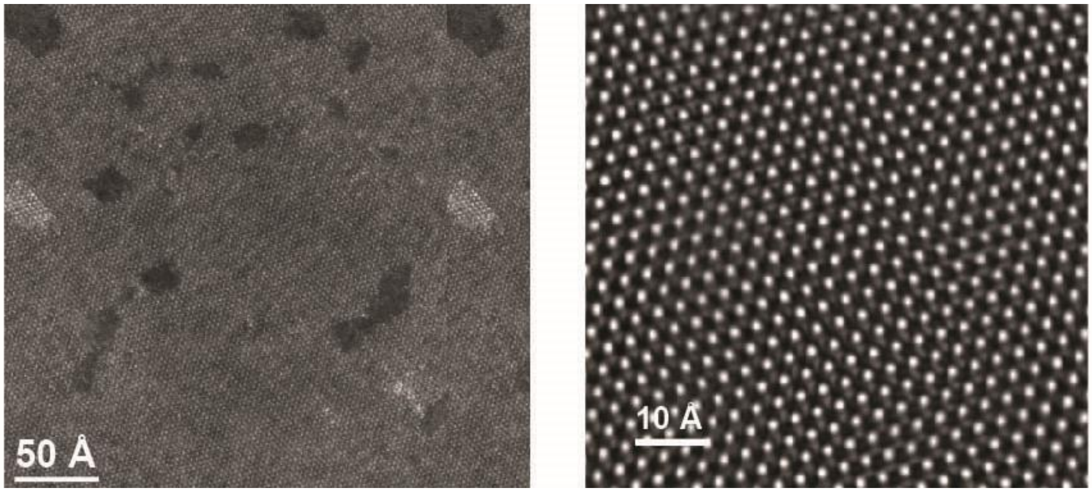

Supplementary Fig. 4. (Left) MoS_2 sheet after heating at 275 ^oC under Ar atmosphere, for 45 m. (Right) High resolution image of the annealed lattice, showing reversion to the 2H phase.

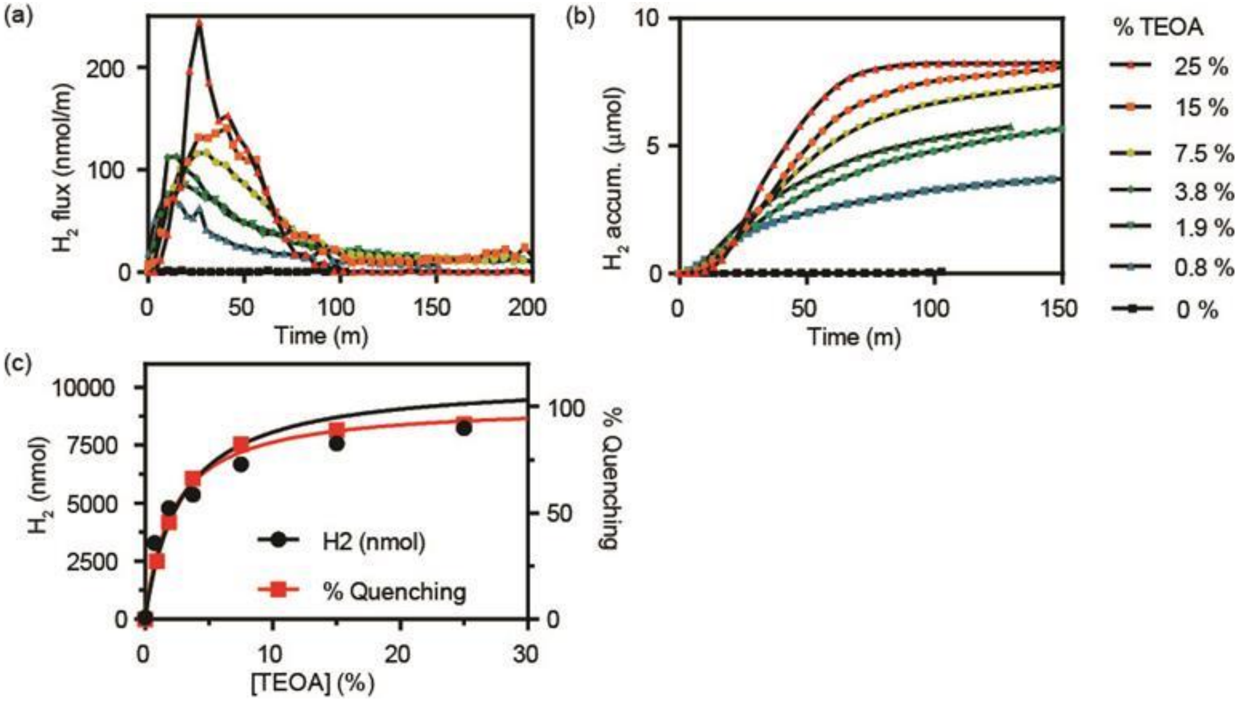

Supplementary Fig. 5. Optimization of catalysis reaction with EY and TEOA. (a) H_2 flux with increasing TEOA concentration. (b) H_2 accumulation with increasing TEOA concentration. (c) overlay of photoquenching of EY with TEOA with H_2 yield, showing good agreement.

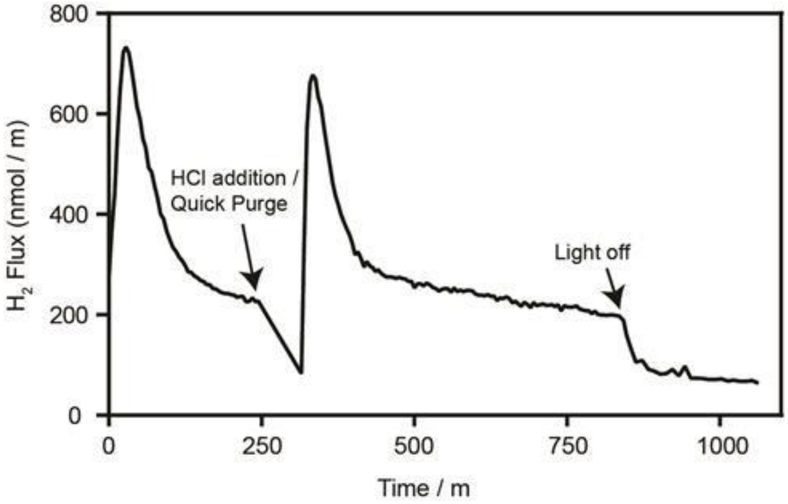

Supplementary Fig. 6. Photocatalytic solution with 40 ppm of MoS_2 was reacted under illumination. Upon waning of reaction, 20 ul of 1M HCl was added, and the mixture was quickly purged. It was observed that with addition of HCl, the reaction can resume, indicating depletion of protons result in HER termination. The reaction also can be terminated by turning off the illumination.

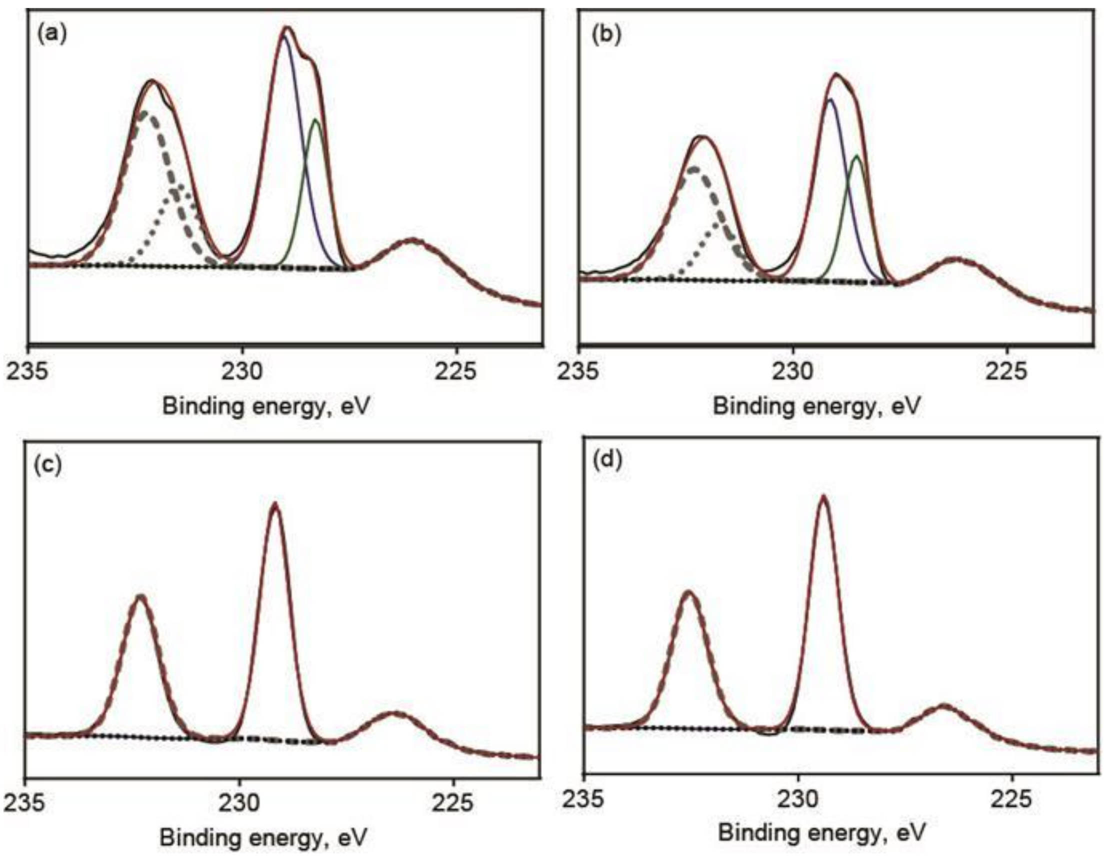

Supplementary Fig. 7. Degree of 1T' transformation was compared using XPS analysis of Mo spectra. Fraction of transformation was determined by integration of shifted peak against control peaks. MoS_2 samples were extracted from reaction vessels after catalysis. (a) MoS2 spectra with 76% transformation using 1 M n-butyl lithium intercalant. (b) 46% transformation from 0.1 M n-butyl Li intercalant, and (c) negligible transformation from 0.01 M n-butyl Li intercalant. (d) Spectra from MoS2 exfoliated with sonication in NMP, without Li intercalant.

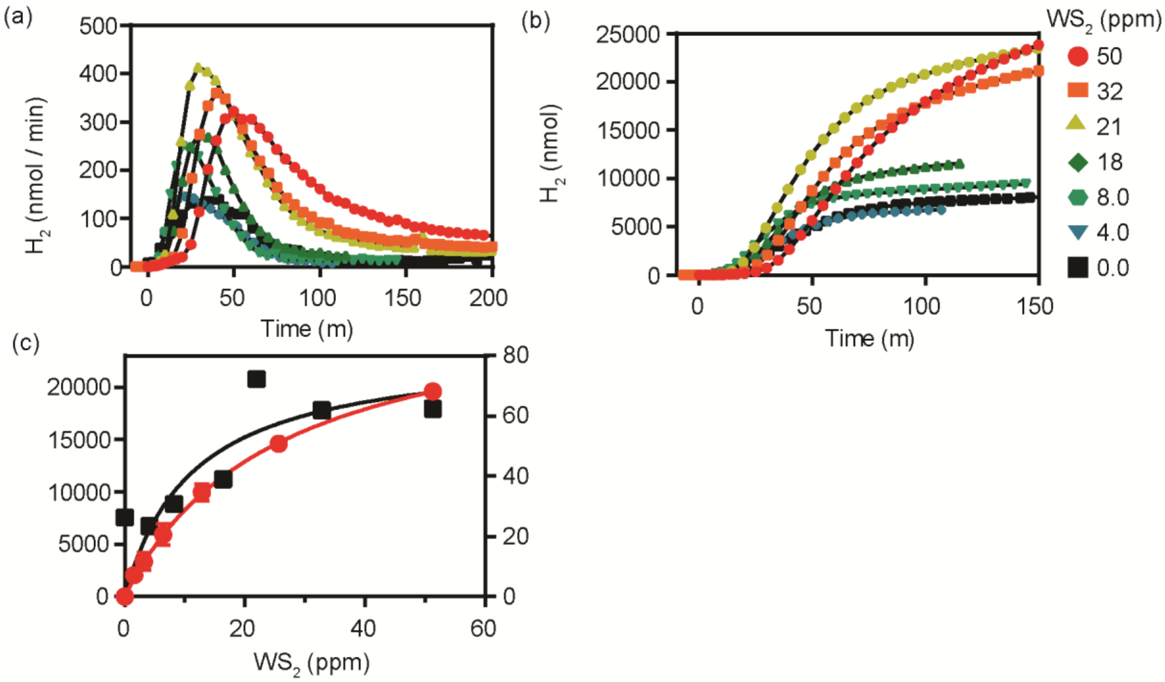

Supplementary Fig. 8. HER reaction with WS_2. (a) H_2 flux with increasing TEOA concentration. (b) H_2 accumulation with increasing TEOA concentration. (c) Overlay of photoquenching of EY with TEOA with H_2 yield.

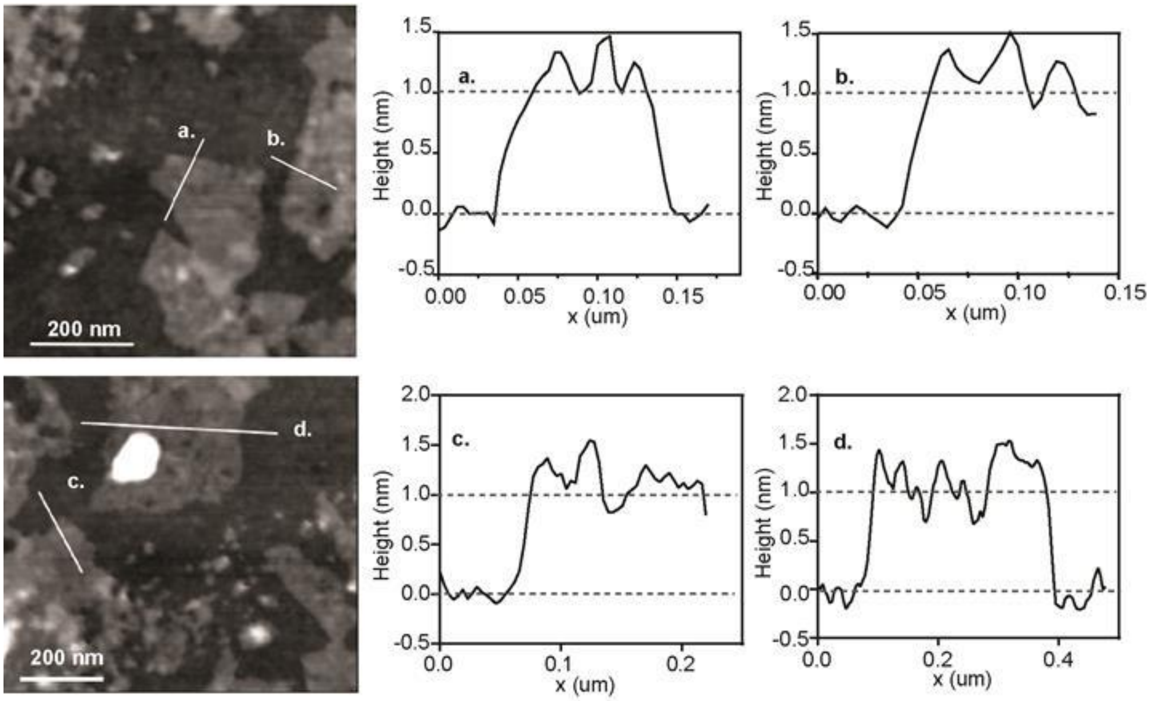

Supplementary Fig. 9. AFM image showing step-height of exfoliated MoS_2 are ≈ 1 nm, consistent with corrugated monolayers.

(a)	TS (eV)	E_{ZPE} (eV)	$\Delta E_{ZPE} - T\Delta S$ (eV)
H_2	0.401	0.271	–
H/1T' MoS_2	0	0.228	0.29
H/2H MoS_2	0	0.198	0.26
H/1T' WS_2	0	0.229	0.29

**(b)**	**Atom**	**x**	**y**	**z**
**1T'**	Mo1	0	0	0
a = 6.545Å	Mo2	0.39543	0.89537	−0.00794
b = 3.176Å	S1	0.1154	0.61504	0.08287
c = 20Å	S2	0.28003	0.27949	-0.09082
α = 90°	S3	0.61968	0.62012	0.06234
β = 90°	S4	0.77575	0.27615	-0.07028
γ = 119.034°				
**2H**	Mo1	0	0	0
a = 6.369Å	Mo2	0.5	0	0
b = 3.1845Å	S1	0.16665	0.66681	0.07808
c = 20Å	S2	0.16669	0.66657	-0.07807
α = 90°	S3	0.66665	0.66681	0.07808
β = 90°	S4	0.66669	0.66657	-0.07807
γ =120°				
**Transition state (saddle)**	Mo1	0	0	0
a = 6.229 Å	Mo2	0.47248	0.90315	-0.03021
b = 3.126 Å	S1	0.1919	0.63148	0.06593
c = 20.368 Å	S2	0.25148	0.29317	-0.09716
α = 84.454°	S3	0.66521	0.62879	0.06312
β = 90.471°	S4	0.73349	0.64082	-0.08711
γ =112.619°				

Supplementary Table 1. (a) Zero point energies and entropies calculated for the construction of the absorption free energies. The entropy and zero point energy of H_2 were calculated using Gaussian 09 [6] and the vibrational frequencies of the H adsorbed states were calculated using VASP. (b) Calculated atomic positions in direct coordinates and lattice parameters for the initial state (1T'), the final state (2H) and the transition state.

1T' Fraction	H_2 Flux (nmol/min)	H_2 Yield (μmol)
c.a. 0.76	698	25.2
c.a. 0.46	491	16.2
c.a. 0	156	6.2

ΔG_H	Θ = 1/16	Θ = 1/8	Θ = 1/4	Θ = 1/2
1T'	0.13	0.16	0.18	0.255
2H	1.6	N/A	N/A	N/A

Supplementary Table 2. (top) Peak H_2 flux and cumulative yield after 100 minutes of reaction as a function of estimated 1T' fraction. (bottom) Gibbs free energy in eV of hydrogen adsorption with increasing hydrogen coverage (Θ).

Supplementary Methods

Lithium intercalation and exfoliation. For MoS_2, lithium intercalation was accomplished by immersing 1 g of MoS_2 powder in 10 ml of 1 M n-butyl lithium. The mixture was stirred vigorously in an inert atmosphere glovebox for 3 to 10 days. After, the compound was washed over three layers of Whatman filter paper (#51, ashless) with 200 ml of hexane and then collected in a bottle. 300 ml of H_2O was then added, and the mixture was sonicated for 3 m to extricate the intercalated MoS_2 powder from the filter paper. The filter paper was then removed from the bottle, and the solution was sonicated for an additional 1.5 h. Unexfoliated portions were removed with centrifugation at 100 g for 3 minutes. The supernatant was then collected, and purified by centrifugation and washing with H_2O (3 x 10,000 g for 1.5 h, followed by resuspension in water after each centrifugation cycle). The solution was then transferred in to a dialysis bag (Fisher Scientific, MW 5000), and dialysed against running water for 3 days. The solution was then again centrifuged at 500 g for 15 m to remove aggregates, and the resultant samples were used as is. For WS_2, Li intercalation was performed at 100 ^oC in a Parr bomb. Exfoliation and purification of WS_2 was the same as MoS_2.

Concentration determination. 50 μl of MoS_2 (or WS_2) was digested with 200 μl of HNO_3 at 75 ^oC overnight. The samples were then diluted to 10 ml using 15 MΩ H_2O. Metal and Lithium concentration were then measured against fixed standards using Atomic Absorbance spectroscopy. In a typical product, Li:Mo molar ratio was found to be 0.012 ± 0.001.

STEM Microscopy and Analysis. A FEI Titan G2 80-200 S/TEM with a Cs probe corrector operated at 200 kV was used in this study. High-angle annular dark-field (HAADF) S/TEM images were acquired with an electron probe size of 0.8 Å, convergence angle of 18.1 mrad, and current of ~100 pA and using an annular detector with a collection range of 60-160 mrad. The high-resolution HAADF images typically are taken at a magnification of 1800kx which gives a

pixel-size of about 0.23 Å or frame-size of 48 nm x 48 nm for 2048 x 2048 pixel frame. Such conditions give rise to an equivalent electron dose rate of ~2800 electrons $Å^{-2}$ s^{-1}. Acquisition of the HAADF image (frame of 2048 pixels × 2048 pixels) takes ∼40 s at a dwell time of ∼10 μs/pixel. In subfigures of Figures 1, 3 and 4, the filtered images were obtained by Fast Fourier transformation (FFT) of the HAADF images into reciprocal space, forming FFT patterns. Reciprocal spots from the patterns (typically, {100} type of reflections) were selected, masked with a 60 pixels filter, and then transformed into real space with inverse FFT. Gatan Digital Micrograph was used for the image processing.

STEM Image Simulation Procedure: The high-resolution S/TEM simulations were carried out using the multi-slice simulation software provided by Cornell University (Earl J. Kirkland, Advanced computing in electron microscopy, Springer, 2nd edition, 2010). For the simulation, a super cell of approximately 50 Å by 50 Å was built using the coordinates supplied in Fig. 2. The pixel sampling used for the supercell was 2048 by 2048. The electron optical parameters used in the simulation are consistent with experimental conditions. They include electron energy of 200 keV, spherical aberration coefficient of 0.1 mm, and defocus of -18nm (under focus), HAADF detector range of 60-160 mrad. Exact atomic positions used in the simulation are restated below from Fig. 2 and Supplementary Table S2 for convenience.

Phase: 2H

Lattice parameters

a (Å)	b (Å)	c (Å)	α (°)	β (°)	γ (°)
3.18448	3.18446	20	90	90	120.0002

Unit-cell volume = 175.644038 $Å^3$

Structure parameters

Atom	x	y	z	Occ.*
Mo	0.33332	0.66669	0.0779	1
S	0.66662	0.33349	0.15598	1
S	0.6667	0.33326	0.99983	1

* Occupancy (Occ.) values are set to 1 when fully occupied.

Phase: 1T

Lattice parameters

a (Å)	b (Å)	c (Å)	α (°)	β (°)	γ (°)
6.44051	6.40723	20	90	90	119.4979

Unit-cell volume = 174.376363 $Å^3$

Structure parameters

Atom	x	y	z	Occ.
Mo	0.99999	0.00004	0.10323	1
S	0.66663	0.33333	0.02327	1
S	0.33334	0.66675	0.18325	1

Phase: 1T'

Lattice parameters

a	b	c	α (°)	β (°)	γ (°)
6.54524	3.17617	20	90	90	119.0337

Unit-cell volume = 363.527288 $Å^3$

Structure parameters

Atom	x	y	z	Occ.
Mo	0	0	0	1
Mo	0.39543	0.10463	0.99205	1
S	0.1154	0.38496	0.08287	1
S	0.28003	0.72051	0.90918	1
S	0.61968	0.37988	0.06234	1
S	0.77574	0.72385	0.92972	1

Phase: 1T"

Lattice parameters

a (Å)	b (Å)	c (Å)	α (°)	β (°)	γ (°)
6.44051	6.40723	20	90	90	119.4979

Unit-cell volume = 718.334526 $Å^3$

Structure parameters

Atom	x	y	z	Occ.
Mo	0.98569	0.48329	0.00219	1
Mo	0.02968	0.00715	0.99957	1
Mo	0.45875	0.43714	0.99954	1
Mo	0.45966	0.00584	0.99945	1
S	0.14933	0.3182	0.91822	1
S	0.14979	0.81375	0.9185	1
S	0.31534	0.15003	0.08808	1
S	0.32171	0.65336	0.07485	1
S	0.65117	0.81641	0.93232	1
S	0.65354	0.3183	0.91879	1
S	0.81263	0.14377	0.07442	1
S	0.81265	0.65278	0.0741	1

Photocatalysis procedure. Stock mixtures of 30% v/v TEOA and 10 mg/ml Eosin Y was freshly prepared for each reaction. MoS_2 (and WS_2) were prepared at twice the desired final concentration (e.g. 80 ppm). To prepare the reaction mixture, 35 ml of the 30% v/v TEOA was mixed with 35 ml of the MoS_2 or WS_2, reaching the final concentration of 15% v/v TEOA and the predetermined MoS_2/WS_2 concentration (e.g. 40 ppm). The mixture was then transferred to a 250 ml two neck flask, to which 1 ml of the 10 mg/ml Eosin Y was added. The mixture was the

covered in aluminum foil, stirred, and purged with continuous Ar flow (20 ccm) for 20 minutes. After Ar purge, aluminum foil covering was removed, and the mixture was illuminated at 1 sun. With the Ar flow kept at 20 ccm, gas samples were continuously monitored at 90 second interval using an Inficon 3000 micro GC gas analyzer until reaction termination.

Computational details. All density functional calculations were carried out using the Vienna *ab-initio* simulation package (VASP)[1] with *plane wave* basis set and projector augmented-wave pseudopotentials (PAW).[2] All energies were calculated with Perdew−Burke−Ernzerhof (PBE) exchange-correlation potentials.[3] For selected structures, we applied the hybrid HSE06 functional[4] to calculate the electronic band gap. The MS_2 (M = Mo, W) monolayers were modeled using surface supercells separated in the periodic direction by a 20 Å thick vacuum slab. We applied a plane wave energy cutoff of 600 eV and Γ−centered 24×24×1, 12×24×1, 12×12×1, and 6×6×1 k-points grids for Brillouin zone sampling of the 1×1 unit cell (2H and 1T), and the 2×1 (1T'), 2×2 (1T''), and 4×4 (H/MS_2) supercells, respectively. The criteria of convergence for energy and force were set as 10^{-4} eV and $3x10^{-3}$ eV/Å.

The hydrogen adsorption energies were calculated using the 4×4 surface supercell containing 16 Mo atoms and 32 S atoms. A dipole correction was applied to cancel the electrostatic interaction between the periodic slabs. The DFT binding energies were calculated as $\Delta E_H = \frac{1}{n}\left(E(surf + nH) - E(surf) - \frac{n}{2}E(H_2)\right)$, where E(surf+nH), E(surf), and $E(H_2)$ are the total energies for the MoS_2 surface with *n* hydrogen atoms adsorbed, the clean MoS_2 surface, and the molecular hydrogen in the gas phase, respectively. The most stable H binding site in the basal plane of MoS_2 is on the top of the S atoms. We define H coverage as the ratio of the number of H and Mo atoms in the basal plane. For 1T' MoS_2, we obtained ΔE_H binding energies of –0.16 eV, –0.13 eV, and –0.11 eV, and –0.035 eV at the 1/16H, 1/8H, 1/4H, and 1/2H coverage. For the 4×4 supercell, this corresponds to n = 1, 2, 4, and 8, respectively.

The adsorption free energy was calculated by adding a thermal correction to the binding energy $\Delta G_H = \Delta E_H + \Delta E_{ZPE} - T\Delta S_H$, where ΔE_{ZPE} and $T\Delta S_H$ are respectively, the differences in the zero point energy and entropy contribution between the H adsorbed state and H_2 in the gas phase. We took $\Delta S_H = -½\ S(H_2)$, where ½ $S(H_2)$ is the entropy of ½ H_2 in the gas phase at standard conditions (T = 298.15 K, Pressure = 1atm). We used the assumption that the vibrational entropy in the adsorbed state is small.[5] For H/1T' MoS_2, E_{ZPE} = 0.228 eV (vibrational frequencies 2530.1 cm^{-1}, 637.6 cm^{-1}, 519.8 cm^{-1}) and E_{ZPE} = 0.271 eV for H_2. With these values, the Gibbs free energy was calculated as $\Delta G_H = \Delta E_H + 0.29$ eV.

Generalized solid-state nudged elastic band (G-SSNEB) calculations were carried out to calculate the activation energy barrier between 1T'-MoS_2 and 2H-MoS_2 shown in Supplementary Figure S1.[7] Further refining calculation using the dimer method made no changes to the energy of the saddle point from the G-SSNEB method.[8] We froze the first Mo atom at (0,0,0) for all the configurations to eliminate a net translation in the optimization.

Supplementary References

1. Kresse G, Furthmüller J. Efficient iterative schemes for \textit{ab initio} total-energy calculations using a plane-wave basis set. *Phys Rev B* **54**, 11169-11186 (1996).

2. Blöchl PE. Projector augmented-wave method. *Phys Rev B* **50**, 17953-17979 (1994).

3. Perdew JP, Burke K, Ernzerhof M. Generalized Gradient Approximation Made Simple. *Phys Rev Lett* **77**, 3865-3868 (1996).

4. Heyd J, Scuseria GE, Ernzerhof M. Hybrid functionals based on a screened Coulomb potential. *J Chem Phys* **118**, 8207-8215 (2003)

5. Nørskov JK, *et al.* Trends in the Exchange Current for Hydrogen Evolution. *J Electrochem Soc* **152**, J23-J26 (2005).

6. Gaussian 09 RD, M. J. Frisch, G. W. Trucks, H. B. Schlegel, G. E. Scuseria, M. A. Robb, J. R. Cheeseman, G. Scalmani, V. Barone, B. Mennucci, G. A. Petersson, H. Nakatsuji, M. Caricato, X. Li, H. P. Hratchian, A. F. Izmaylov, J. Bloino, G. Zheng, J. L. Sonnenberg, M. Hada, M. Ehara, K. Toyota, R. Fukuda, J. Hasegawa, M. Ishid a, T. Nakajima, Y. Honda, O. Kitao, H. Nakai, T. Vreven, J. A. Montgomery, Jr., J. E. Peralta, F. Ogliaro, M. Bearpark, J. J. Heyd, E. Brothers, K. N. Kudin, V. N. Staroverov, R. Kobayashi, J. Normand, K. Raghavachari, A. Rendell, J. C. Burant, S. S. Iyengar, J. Tomasi, M. Cossi, N. Rega, J. M. Millam, M. Klene, J. E. Knox, J. B. Cross, V. Bakken, C. Adamo, J. Jaramillo, R. Gomperts, R. E. Stratmann, O. Yazyev, A. J. Austin, R. Cammi, C. Pomelli, J. W. Ochterski, R. L. Martin, K. Morokuma, V. G. Zakrzewski, G. A. Voth, P. Salvador, J. J. Dannenberg, S. Dapprich, A. D. Daniels, Ö. Farkas, J. B. Foresman, J. V. Ortiz, J. Cioslowski, and D. J. Fox, Gaussian, Inc., Wallingford CT, 2009. .

7. Sheppard D, Xiao P, Chemelewski W, Johnson DD, Henkelman G. A generalized solid-state nudged elastic band method. *J Chem Phys* **136**, 074103 (2012).

8. Xiao P, Sheppard D, Rogal J, Henkelman G. Solid-state dimer method for calculating solid-solid phase transitions. *J Chem Phys* **140**, 174104 (2014).
